# Discovery of *N*-(2-Aminophenyl)-4-(bis(2-chloroethyl)amino)Benzamide as a Potent Histone Deacetylase Inhibitor

**DOI:** 10.3389/fphar.2019.00957

**Published:** 2019-08-30

**Authors:** Lihui Zhang, Xiaoyang Li, Yiming Chen, Minghui Wan, Qixiao Jiang, Li Zhang, C. James Chou, Weiguo Song, Lei Zhang

**Affiliations:** ^1^School of Stomatology, Weifang Medical University, Weifang, China; ^2^School of Medicine and Pharmacy, Ocean University of China, Qingdao, China; ^3^Department of Medicinal Chemistry, School of Pharmacy, Weifang Medical University, Weifang, China; ^4^School of Public Health, Qingdao University, Qingdao, China; ^5^School of Pharmacy, Qingdao University, Qingdao, China; ^6^Department of Drug Discovery and Biomedical Sciences, College of Pharmacy, Medical University of South Carolina, Charleston, SC, United States

**Keywords:** HDAC, inhibitor, nitrogen mustard, antitumor, selectivity

## Abstract

Inhibition of histone deacetylases (HDACs) has been an important emerging therapy for the treatment of multiple cancers. However, the application of HDAC inhibitors is restricted by the limited potency against solid tumors. In order to discover novel HDAC inhibitors with potent antitumor activities, nitrogen mustard group was introduced to the structure of CI994. The derived molecule *N*-(2-aminophenyl)-4-(bis(2-chloroethyl)amino)benzamide (NA) exhibited enzyme inhibitory pattern of class I selectivity with IC_50_ values of 95.2, 260.7, and 255.7 nM against HDAC1, HDAC2, and HDAC3, respectively. In the antiproliferative assay, NA exhibited 10.3-fold (2.66 μM) and 11.3-fold (1.73 μM) higher potency than did suberoylanilide hydroxamic acid (SAHA) (27.3 and 19.5 μM) in inhibition of A2780 and HepG2 cell growth, respectively. Further HepG2 cell-based cell cycle and apoptosis studies revealed that induction of the G2/M phase arrest and cell apoptosis contributes to the antitumor effects of NA. It is suggested that NA could be utilized as a lead compound in the development of bifunctional HDAC inhibitors for the treatment of solid tumors.

## Introduction

Histone deacetylases (HDACs) are a family of enzymes that are responsible for the epigenetic regulation of histone and more than 50 nonhistone proteins (Bernstein et al., 2000; De Ruijter et al., 2003; Foglietti et al., 2006). Currently, four classes of HDACs including 18 different isoforms have been identified in human (Zhang et al., 2015). Class I HDACs contain HDAC1, 2, 3 and 8, which mostly exist in the nucleus. Class II HDACs shuttle between the cytoplasm and the nucleus and are subdivided into IIa (HDAC4, 5, 7, and 9) and IIb (HDAC6 and 10). Class III HDACs known as sirtuins (sirt1–7) are a group of NAD^+^-dependent enzymes. HDAC11 classified as class IV is low homologous with either class I or class II HDACs. In contrast with class III, class I, II, and IV HDACs are zinc-dependent HDACs with zinc ions in their catalytic sites.

HDAC inhibitors (HDACIs) have been developed for treatment of epigenetic disorders, especially cancer (Marks et al., 2001). Suberoylanilide hydroxamic acid (SAHA) (Richon et al., 1998) and FK228 (Ueda et al., 1994) exhibited therapeutic significance for the treatment of refractory cutaneous T-cell lymphoma (CTCL) and had been approved by US Food and Drug Administration (FDA). Additionally, PDX101 (Yang et al., 2010) and LBH589 (Neri et al., 2012) had been approved for the treatment of peripheral T-cell lymphoma (PTCL) and multiple myeloma, respectively. Chidamide (Dong et al., 2009) is a benzamide HDACI approved by Chinese Food and Drug Administration (CFDA) for the treatment of relapsed or refractory PTCL in 2015. Moreover, there are also more than 15 HDACIs currently being evaluated in clinical investigations for the treatment of tumor and other diseases, administrated in single and combination doses (http://www.clinicaltrials.gov ).

The discovery of nitrogen mustard as an alkylating agent initiated modern cancer chemotherapy in 1942. The alkylating agents inhibit tumor by crosslinking two DNA strands, preventing DNA replication and inducing cell death (Singh et al., 2018). The relative lack of selectivity towards DNA of both tumor cell and normal cell led to nitrogen mustard’s various adverse side effects along with therapeutic effects. The aromatic nitrogen mustards generated by replacing the methyl group in nitrogen mustard with aromatic groups had been characterized with stabilized nitrogen and reduced drug reactivities.

**Figure 1:**
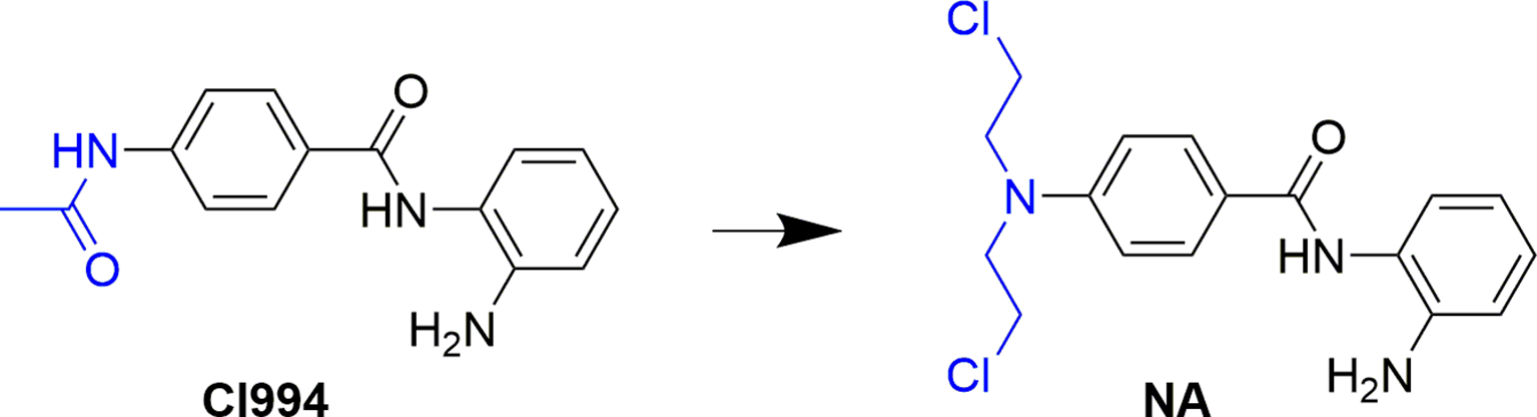
Design of NA from the structure of CI994.

Development of bifunctional molecules by linking of aromatic nitrogen mustards to the structure of targeted antitumor agents is a promising strategy in anticancer drug development (Chen et al., 2018). In the discovery of effective and safe antitumor molecules, the aromatic nitrogen mustard fragment was employed to the structure of HDAC inhibitor in the present study. First, molecular design was performed by replacement of the acetyl group of CI994, a potent HDACI being evaluated in phase II clinical trials to a 4-(bis(2-chloroethyl)amino)benzoyl group (**Figure 1**). The derived target molecule, *N*-(2-aminophenyl)-4-(bis(2-chloroethyl)amino)benzamide (NA), was synthesized and evaluated with enzymatic selectivity assay, *in vitro* cancer cell-based screening, cell cycle, and apoptosis studies.

### Chemistry

**Scheme 1:**
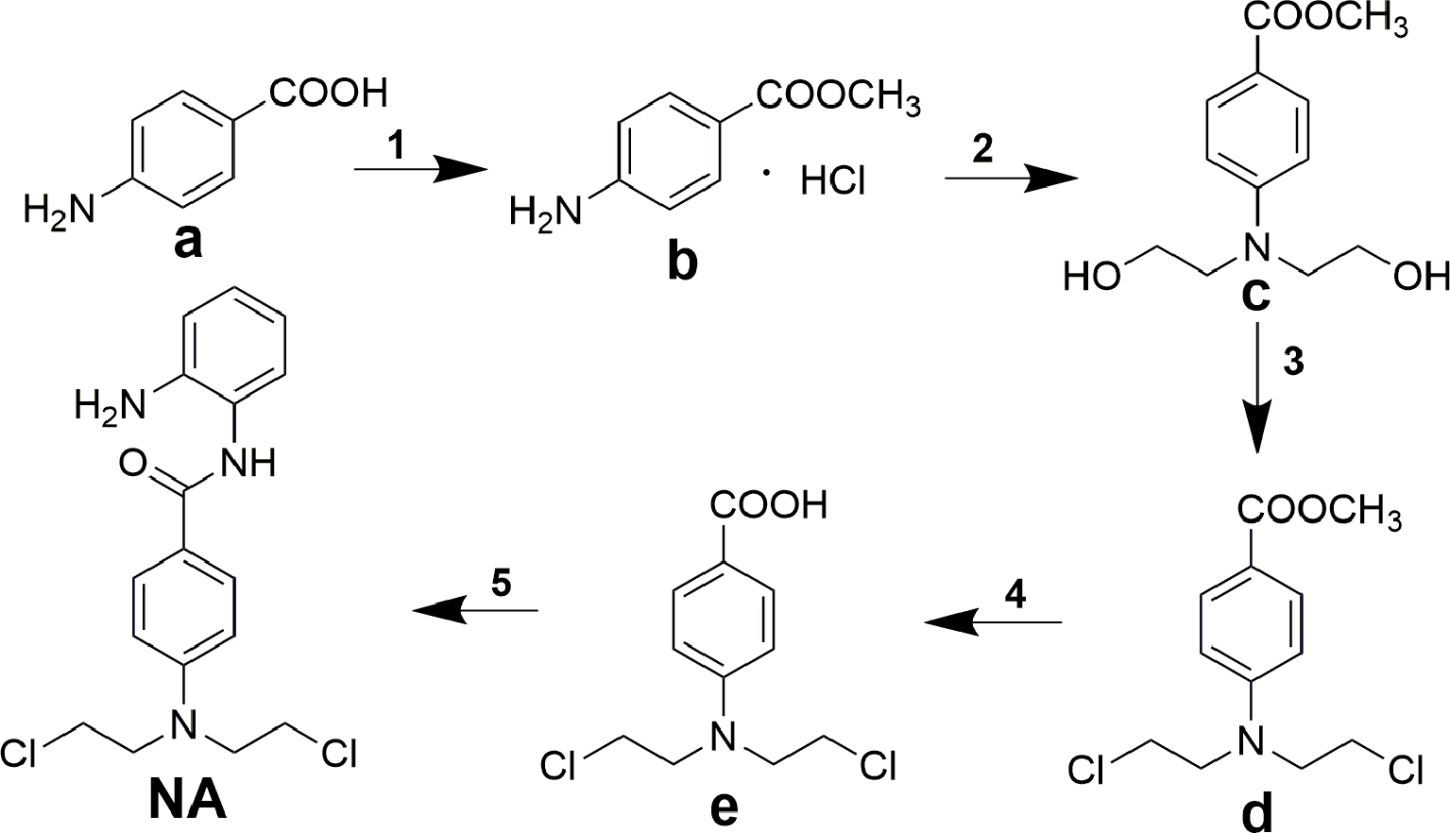
Synthesis of compound NA. Reagents and conditions: (**1**) acetyl chloride, methanol; (**2**) ethylene oxide, acetic acid/water; (**3**) POCl_3_, toluene; (**4**) concentrated hydrochloric acid; (**5**) *N*,*N*′-carbonyldiimidazole, trifluoroacetic acid, tetrahydrofuran.

Title molecule *N*-(2-aminophenyl)-4-(bis(2-chloroethyl)amino)benzamide (NA) was synthesized according to the procedures described in **Scheme 1**. The starting material 4-aminobenzoic acid (a) was protected by methyl esterification, and the intermediate methyl 4-(bis(2-hydroxyethyl)amino)benzoate (c) that followed was derived by addition of two 2-hydroxyethyl groups using ethylene oxide. Subsequent substitution of chlorine for hydroxyl group and carboxyl group deprotection afforded key intermediate 4-(bis(2-chloroethyl)amino)benzoic acid (e). Target compound NA was derived by condensation of **e** with *o*-phenylenediamine.

## Results and Discussions

### Enzyme Inhibitory Selectivity of NA

**Figure 2:**
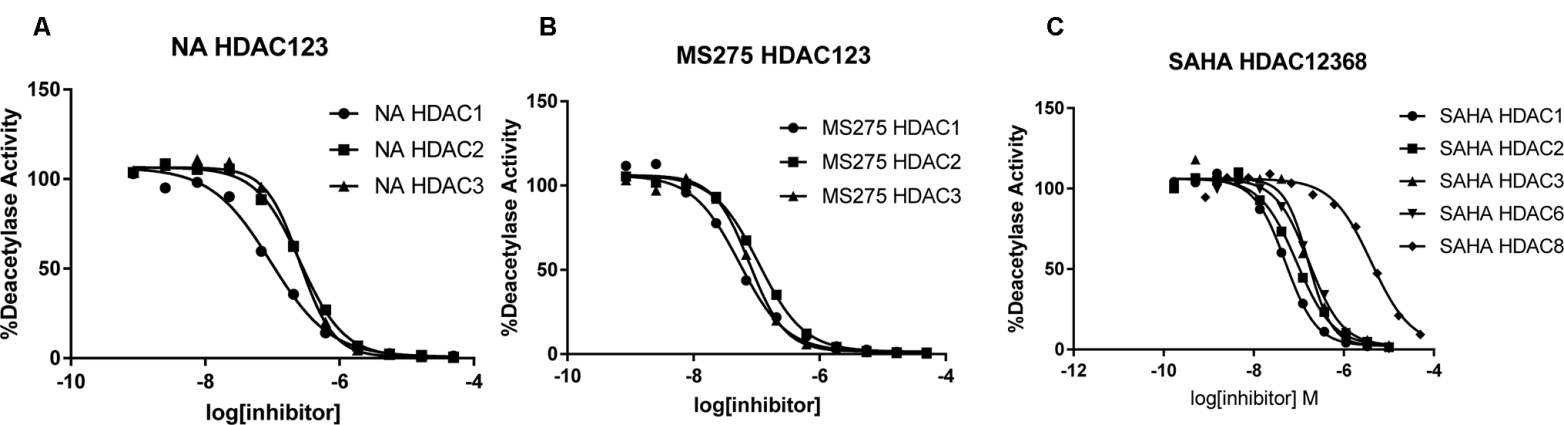
Inhibition curves of NA **(A)**, MS275 **(B)**, and SAHA **(C)** against various HDACs.

To determine the isoform selectivity of NA, an enzymatic activity inhibition assay was performed against HDAC1, 2, 3, 4, 6, 7, 8, and 9 (**Figure 2**). Compared with the nonselective inhibitor SAHA and the class I selective MS275, NA also exhibited inhibitory selectivity of class I HDACs with IC50 values of 95.2, 260.7, and 255.7 nM against HDAC1, HDAC2, and HDAC3, respectively (**Table 1**). Nevertheless, NA exhibited much reduced inhibitory activity against HDAC8, which also belongs to the class I HDACs with IC50 value of over 5,000 nM. The IC50 values of NA in inhibition of the class IIa HDAC4, HDAC7, and HDAC9 and the IIb HDAC6 were also over 5,000 nM, indicating high selectivity of NA. CI994 was also reported to be a class I HDAC inhibitor. However, in the subfamily composed of HDAC1, 2 and 3, CI994 was nonselective between HDAC1 (41 nM) and HDAC3 (46 nM), while it had relatively weak HDAC2 inhibitory activity (147 nM). It is remarkable that NA is an HDAC1-selective inhibitor with 2.74-fold selectivity versus HDAC2 and 2.69-fold selectivity versus HDAC3, respectively. Therefore, molecule NA could be promising in the treatment of a specific disease by targeting HDAC1 and in probing the functions of HDAC1 in the disease development.

**Table 1:** Enzyme inhibitory activity of NA compared with MS275 and SAHA (IC_50_, nM)^a^.

HDACIs	HDAC1	HDAC2	HDAC3	HDAC4	HDAC6	HDAC7	HDAC8	HDAC9
NA	95.2	260.7	255.7	>5,000	>5,000	>5,000	> 5,000	>5,000
MS275	53.9	108.2	77.2	>5,000	>5,000	>5,000	> 5,000	>5,000
SAHA	50.7	90.4	164.1	>5,000	169.5	>5,000	4,008	>5,000
^a^Assays were performed in replicate (n ≥ 2); the SD values are <10% of the mean.								

### Antiproliferative Activity of NA

A series of tumor cell lines including the lung cancer A549 and Calu-3; breast cancer MDA-MB-231, MCF-7, and MDA-MB-468; colon carcinoma LoVo and Colo205; ovarian cancer A2780 and SKOV3; liver cancer HepG2; gastric cancer MKN45; pancreatic cancer PNAC-1 cells; and leukemic U937 cells were cultured for the antiproliferative assay of NA. Against most of the tested cell lines, NA exhibited limited inhibitory activity than did SAHA (**Table 2**). Remarkably, in the inhibition of the growth of A2780 and HepG2 cells, NA was revealed to be 10.3-fold (IC50 values of 2.66 μM) and 11.3-fold (IC50 values of 1.73 μM) more potent than was SAHA with IC50 values of 27.3 and 19.5, respectively. The results suggested that NA may be further specifically used for the treatment of liver cancer and ovarian cancer. Since the application of classical HDACIs had been restricted by the lack of therapeutic efficacy against solid tumors, the discovery of NA, an HDAC1-selective inhibitor with potent solid tumor (ovarian and liver cancer) inhibitory effects, is remarkable.

**Table 2:** Antiproliferative activity of NA compared with SAHA (IC_50_, μM)^a^.

Cell line	Tumor type	NA	SAHA
A549	Lung cancer	14.74	1.73
Calu-3	Lung cancer	5.81	3.18
MDA-MB-231	Breast carcinoma	7.64	1.11
MCF-7	Breast carcinoma	18.71	2.78
MDA-MB-468	Breast carcinoma	6.33	1.44
LoVo	Colon carcinoma	10.37	1.07
Colo205	Colon carcinoma	33.81	2.01
A2780	Ovarian cancer	2.66	27.3
SKOV3	Ovarian cancer	3.91	1.73
HepG2	Liver cancer	1.73	19.5
MKN45	Gastric Cancer	14.35	16.06
PNAC-1	Pancreatic cancer	8.52	6.57
U937	Myeloid leukemia	1.21	1.02
^a^Assays were performed in replicate (n ≥ 2); the SD values are <10% of the mean.			

### Cell Cycle Analysis

**Figure 3:**
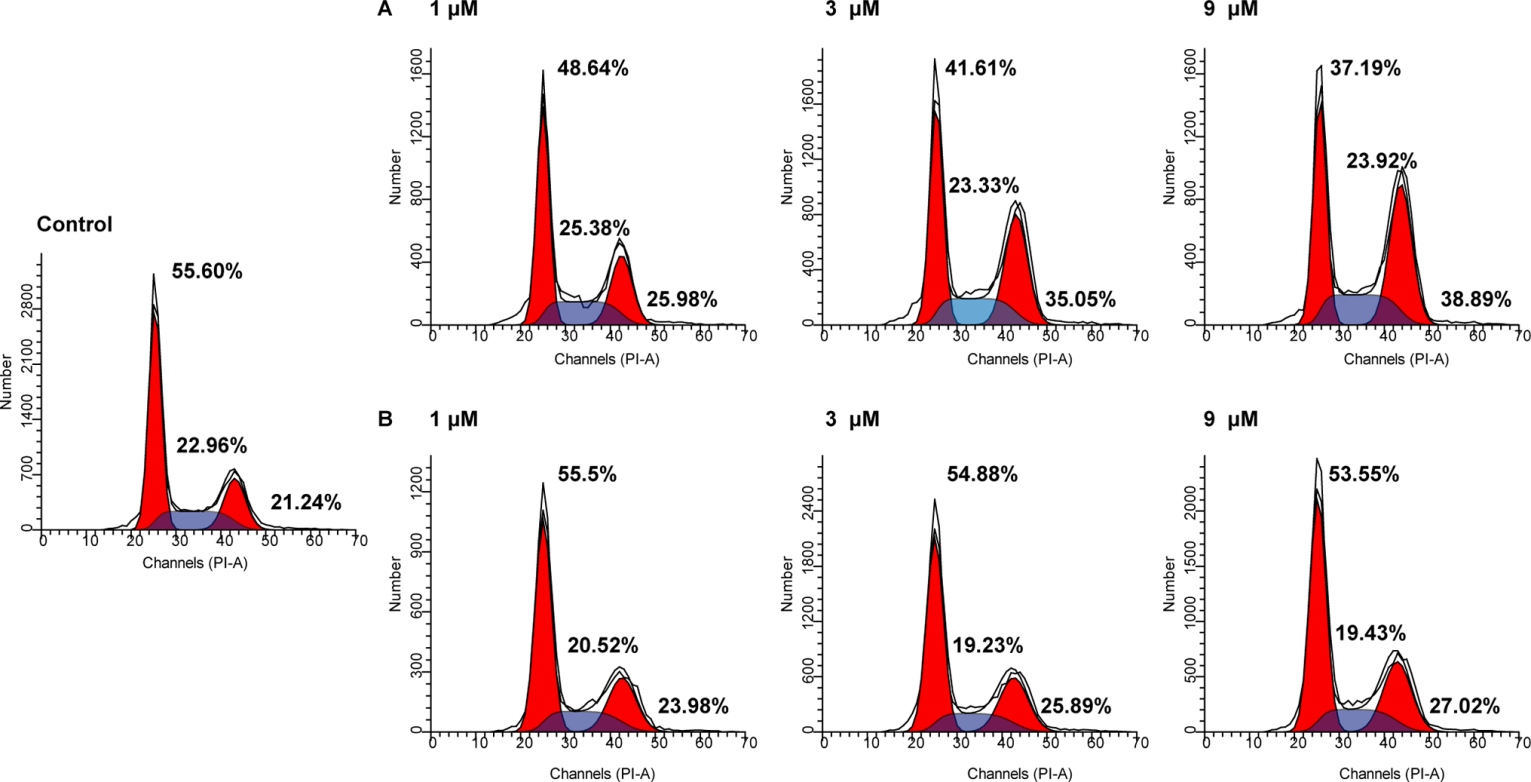
Cell cycle analysis of molecule NA on HepG2 cells. Cells were treated with NA **(A)** and SAHA **(B)** at concentrations of 1, 3, and 9 µM for 6 h. The results were detected by flow cytometry analysis.

The cell cycle consists of three distinct phases including the G0/G1 phase, S phase, and G2/M phase. In tumor cells, as a result of genetic mutations, dysregulated cell cycle resulted in uncontrolled cell proliferation. To evaluate the effects of NA on cell cycle distributions, HepG2 cells were treated with various doses of NA and SAHA (1, 3, and 9 µM) for 6 h. As shown in **Figure 3**, both NA (**Figure 3A**) and SAHA (**Figure 3B**) could regulate cell cycle in a dose-dependent manner. It is revealed that NA treatment leads to significantly higher accumulation of HepG2 cells at the G2/M phase (25.98%, 35.05%, and 38.89% at concentrations of 1, 3, and 9 µM, respectively) compared with SAHA (23.98%, 25.89%, and 27.02% at concentrations of 1, 3, and 9 µM, respectively). At the same time, reduced cell population at the G0/G1 phase was detected from 55.60% (control) to 48.64% (1 µM), 41.61% (3 µM), and 37.19% (9 µM) following treatment with NA, while SAHA treatment had no significant effects in the population of HepG2 cells at the G0/G1 phase. It is suggested that induction of the G2/M phase arrest contributes to the antiproliferative effects of molecule NA.

### Cell Apoptosis Assay

**Figure 4:**
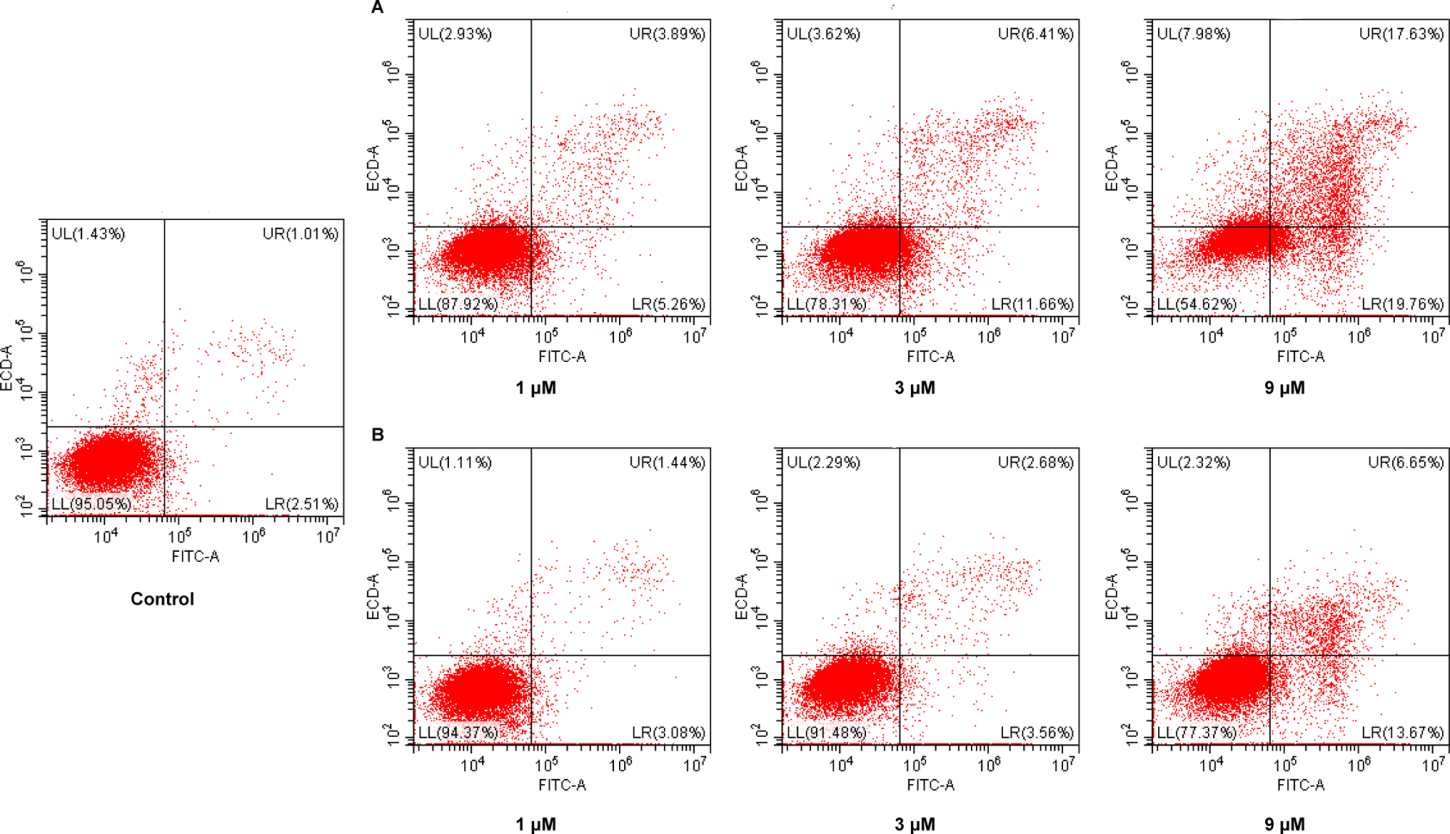
Pro-apoptotic effect of molecule NA. HepG2 cells were treated with NA **(A)** and SAHA **(B)** at concentrations of 1, 3, and 9 µM for 24 h. Then cells were stained with annexin V-FITC/PI, and the results were detected by flow cytometry analysis.

Cancer is one of the scenarios where too little apoptosis occurs, resulting in malignant cells that will not die. Therefore, apoptosis induction plays an important role in the treatment of cancer. To further investigate the role of apoptosis in the antitumor effect of NA, flow cytometry analysis was performed by staining HepG2 cells with annexin V-fluorescein isothiocyanate (FITC)/propidium iodide (PI). It is revealed that both NA (**Figure 4A**) and SAHA (**Figure 4B**) treatment induced HepG2 cell apoptosis in a dose-dependent manner. After treatment with difference doses of NA (1, 3, and 9 µM), the percentage of apoptotic cells was significantly increased from 3.52% of the control to 9.15%, 18.07%, and 37.39%, respectively, than that of SAHA (4.52%, 6.24%, and 20.32% at concentrations of 1, 3, and 9 µM, respectively). It is indicated that induction of cell apoptosis contributes to the antitumor effect of NA.

### Western Blotting studies

**Figure 5:**
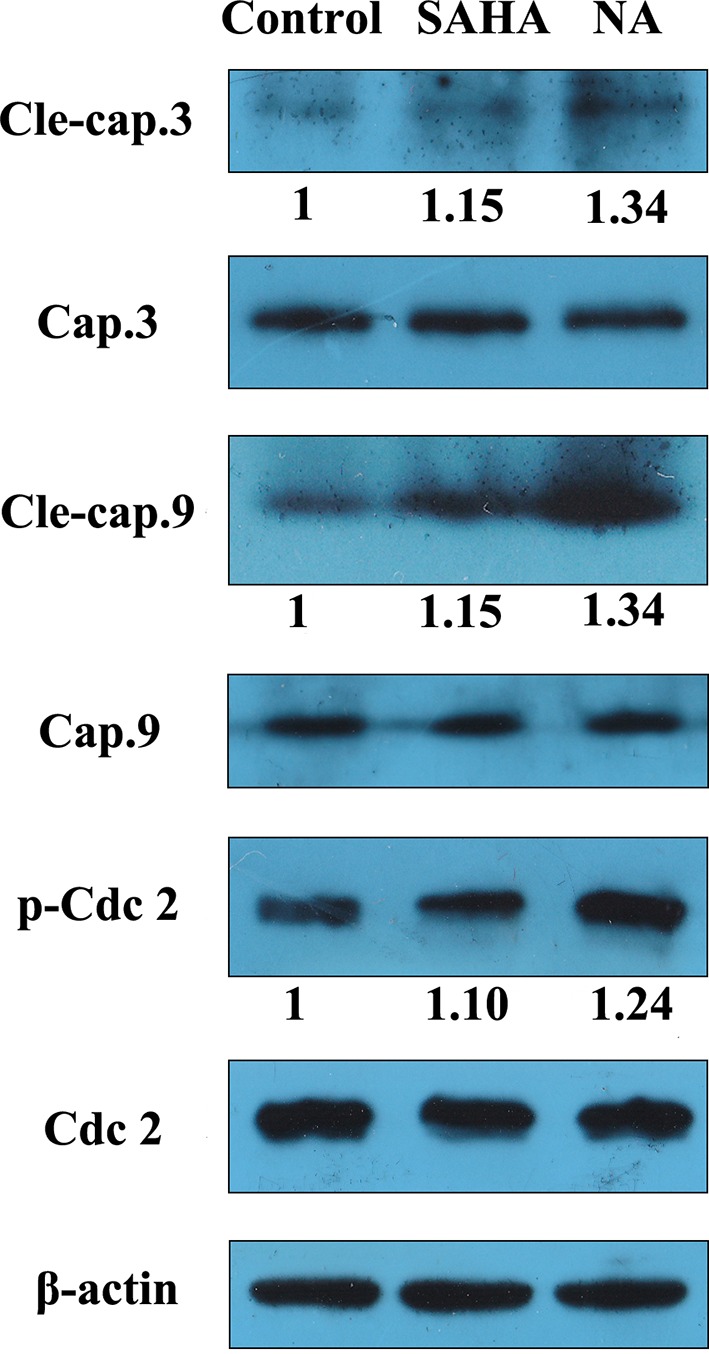
NA induced apoptosis-related protein expression in HepG2 cell. HepG2 cells were treated with SAHA and NA at 1 µM for 24 h. The apoptosis-related proteins cleaved caspase-3, caspase-9, and cell cycle-related protein cdc2 were analyzed by western blotting. β-Actin was used as an internal control. A representative immunoblot from three independent experiments giving similar results was shown for each western blot experiment. Densitometry was performed using AlphaEaseFC-v4.0.0 program.

Western blotting analysis was performed to evaluate effects of NA on the expression of apoptosis and cell cycle-related proteins in tumor cells. Sequential activation of caspases such as caspase-3 and caspase-9 plays a central role in apoptosis. Cdc2 is a specific regulator in the cell cycle transition from the G2 to M phase. Therefore, effects of NA treatment on the protein expression levels of caspase-3, cleaved caspase-3, caspase-9, cleaved caspase-9, cdc2, and phosphorylated cdc2 were evaluated in HepG2 cells (**Figure 5**). The results revealed that NA remarkedly increased the protein expression levels of cleaved caspase-3 and caspase-9 and up-regulated the phosphorylation of cdc2 than did SAHA. It is suggested that NA promoted HepG2 cell apoptosis *via* activation of caspase-3 and caspase-9, and induced the G2/M phase arrest of HepG2 cells *via* activation of cdc2.

## Conclusion and Discussion

In the discovery of HDAC inhibitors with potent antitumor activity, nitrogen mustard group was introduced to the structure of CI994. The derived molecule NA exhibited class I selectivity, and especially HDAC1 inhibitory activity (with IC_50_ values of 95.2 nM) in the enzyme inhibitory assay. In the antiproliferative assay, NA exhibited less potent activity in the inhibition of the growth of most tested cells. However, in the inhibition of A2780 and HepG2 cells, NA exhibited significantly improved activities than did SAHA. Further, HepG2 cell-based cell cycle and apoptosis analysis revealed the role of the G2/M phase arrest and apoptosis in the antitumor effects of NA. Western blotting revealed induction of cleaved caspase 3/9 and phosphorylation of cdc2, further confirming the participation of apoptosis and cell cycle arresting in NA-induced antitumor effects. Collectively, a potent HDAC1 inhibitor (NA) was discovered, which could be utilized as a potent lead compound in the development of anticancer agents targeting solid tumors such as liver cancer.

Inhibition of HDACs is an effective strategy for the treatment of cancer. A large number of HDAC inhibitors have been designed, synthesized, and evaluated in the anticancer activity tests. Until now, four HDAC inhibitors have obtained approval from the US FDA for the treatment of cancer. However, most HDAC inhibitors exhibited limited potency against solid tumors, and none of the approved HDAC inhibitors showed significant potency in clinical trials for the treatment of solid tumors. In the present study, nitrogen mustard group was introduced to the structure of HDAC inhibitor (CI994), the derived molecule that exhibited improved potency in the growth inhibition of solid tumor cells (A2780 and HepG2) compared with SAHA. It is suggested that the insufficient potency of HDACIs against solid tumors could be overcame by development of bifunctional molecules with pharmacophores of other anticancer drugs, such as the nitrogen mustard group.

## Materials and Methods

All commercially available starting materials, reagents, and solvents were used without further purification. All reactions were monitored by thin-layer chromatography (TLC) with 0.25-mm silica gel plates (60GF-254). UV light and ferric chloride were used to visualize the spots. ^1^H NMR and ^13^C NMR spectra were recorded on a Bruker DRX spectrometer at 500 MHz, using tetramethylsilane (TMS) as an internal standard. High-resolution mass spectra were performed in Shandong Analysis and Test Center in Jinan, China. The derived target compound (NA) is of 98.28% purity proved by high-performance liquid chromatography (HPLC) analysis, which was performed on a Waters Acquity H class HPLC instrument using an Inertsil ODS.3 column (150 mm × 4.6 mm). The mobile phase was acetonitrile–water, and linear gradient elution (with H_2_O% from 5% to 90% in 3 min) was used with detection wavelength of 254 nm.

Methyl 4-aminobenzoate hydrochloric acid (**b**) has been synthesized and described in our previous work.

Methyl 4-(bis(2-hydroxyethyl)amino)benzoate (**c**). Methyl 4-aminobenzoate hydrochloric acid (**b**) (18.8 g, 100 mmol) was dissolved in water (50 ml) and glacial acetic acid (50 ml). Ethylene oxide (60 ml) was added with stirring, and the mixture was kept for 24 h at room temperature. The clear yellow solution was poured into water (100 ml), a slight excess sodium bicarbonate was carefully added with stirring, a gummy precipitate was obtained, which was extracted with ethyl acetate and dried over MgSO_4_. The solvent was evaporated and recrystallized to give desired compound **c** (18.2 g, 76% yield). Electrospray ionization–mass spectrometry (ESI-MS) *m*/*z*: 240.3 [M + H]^+^.

Methyl 4-(bis(2-chloroethyl)amino)benzoate (**d**). To a solution of compound **c** (2.40 g, 10 mmol) in toluene (30 ml), phosphorus oxychloride (10 ml) was added and refluxed for 1 h. The solution was evaporated until a clear gummy residue was obtained (**d**). Concentrated hydrochloric acid (6 M) was added to the gummy residue and refluxed for 4 h. The pink solution was filtered, and 4-(bis(2-chloroethyl)amino)benzoic acid (**e**) was obtained by recrystallization of the filtered solid in ethyl acetate (1.78 g, 68% yield). ESI-MS *m*/*z*: 263.1 [M + H]^+^.

*N*-(2-Aminophenyl)-4-(bis(2-chloroethyl)amino)benzamide (NA). To a solution of compound **e** (2.0 g, 7.6 mmol) in tetrahydrofuran (THF) (40 ml), CDI (1.8 g, 11 mmol) was added, and the solution was refluxed for 3 h. *N*,*N*′-Carbonyldiimidazole (6.5 g, 60 mmol) and triﬂuoroacetyl (TFA) were added with stirring, and the mixture was kept for 20 h at room temperature. Then, the solvent was evaporated with the residue being taken up in EtOAc (50 ml). The EtOAc solution was washed with 1 M of citric acid (3 × 20 ml), NaHCO_3_ (3 × 20 ml), and brine (3 × 20 ml); dried over MgSO_4_; and evaporated under vacuum. The desired compound NA (1.35 g, 50% yield) was derived by crystallization in EtOAc as white powder. High-resolution mass spectrometry (HRMS) (AP-ESI) *m*/*z* calcd for C_17_H_20_Cl_2_N_3_O [M + H]^+^ 352.0983, found 352.0979. ^1^H NMR (500 MHz, (CD_3_)_2_SO) δ 9.39 (s, 1H), 7.87 (d, *J* = 8.8 Hz, 2H), 7.14 (d, *J* = 7.6 Hz, 1H), 6.94 (t, *J* = 7.6 Hz, 1H), 6.83 (d, *J* = 8.9 Hz, 2H), 6.77 (d, *J* = 7.8 Hz, 1H), 6.59 (t, *J* = 7.5 Hz, 1H), 4.82 (s, 2H), 3.95–3.66 (m, 8H). ^13^C NMR (101 MHz, DMSO) δ 165.27, 149.38, 143.52, 130.04, 127.00, 126.57, 124.45, 122.63, 116.83, 116.68, 111.38, 52.27, 41.52 ppm. HPLC retention time: 1.96 min, gradient eluted by CH_3_CN/H_2_O.

### *In Vitro* HDAC Inhibitory Assay

All of the HDAC enzymes were bought from BPS Bioscience. In vitro HDAC inhibition assays were conducted as previously described. Briefly, 20 μl of recombinant HDAC enzyme solution (HDAC1−9) was mixed with various concentrations of tested compound (20 μl). The mixture was incubated at 30°C for 1 h (for the time-dependent assay, the mixture was incubated for 15, 30, 60, and 90 min, respectively), and then 10 μl of fluorogenic substrate (Boc-Lys (acetyl)-AMC (3 mM) for HDAC1, 2, 3, and 6, Boc-Lys (trifluoroacetyl)-AMC (3 mM) for HDAC 4, 7, 8, and 9) was added. After incubation at 30°C for 2 h, the catalytic reaction was stopped by addition of 10 μl of developer containing trypsin and trichostatin A (TSA). After 30 min, fluorescence intensity was measured using a microplate reader at excitation and emission wavelengths of 360 and 460 nm, respectively. The inhibition ratios were calculated from the fluorescence intensity readings of tested wells relative to those of control wells, and the IC_50_ curves and values were determined by GraphPad Prism 6.0 software.

### MTT Assay

Antiproliferative activities of NA were evaluated by MTT assay using SAHA as the positive control. The stock solutions of tested compounds were diluted with culture medium. The cells were seeded in 96-well plates at a density 5 × 10^3^ cells per well and incubated until confluency of 90–95%, and then each well was treated with 100 µl of medium containing the desired concentrations of tested compounds and incubated for 48 h. MTT working solution of 20 µl (5 mg/ml) was then added to each well and incubated for another 4 h. At the end of incubation, the medium were carefully removed, and 200 µl of DMSO was added. The optical density at 490 and 630 nm was then measured with a microplate reader (Model 680, Bio-Rad). The percentage of cell growth inhibition was calculated with the following equation: % inhibition = [1 − (Sample group OD_490_ − Sample group OD_630_)/(Control group OD_490_ − Control group OD_630_)] × 100%. The IC_50_ values were calculated with Origin 7.5 software, and standard deviations of the IC_50_ values were obtained from at least three independent experiments.

### Cell Cycle Assay

HepG2 cells in logarithmic growth phase were seeded in 6-well plates (6 × 10^5^ cells/well) and incubated with different doses of NA and SAHA (1, 3, and 9 µM) for 6 h. Cells were then washed twice with cold phosphate-buffered saline (PBS) and fixed in 70% precooled ethanol at 4°C for 12 h. After the fixation, cells were washed again with PBS and stained with PI/RNase A for 30 min at room temperature and eventually subjected to flow cytometry (CytoFLEX, Beckman Coulter) for cell cycle distribution determination.

### Annexin V/PI Detection

HepG2 cells in logarithmic growth phase were seeded in 6-well plates (4×10^5^ cells/well) and incubated with different doses of NA and SAHA (1, 3, and 9 µM) for 24 h. Cells were then washed with PBS, collected, resuspended with binding buffer from the annexin V-FITC kit (Thermo Fisher Co., USA) and then added with 5 µl of annexin V-FITC and mixed gently. After 10 min of incubation, 1 µl of PI was added to each sample and mixed gently. After incubation at room temperature for another 20 min in the dark, cells were subjected to flow cytometry (CytoFLEX, Beckman Coulter).

### Western Blot

After incubation with NA and SAHA for 24 h, cells were rinsed with cold PBS, and then radioimmunoprecipitation assay (RIPA) buffer was added, and protein samples were collected by scrubbing and centrifuged at 14,000*g* for 10 min. Protein sample concentrations were determined with Brandford assay. Thirty micrograms of each sample was subjected to sodium dodecyl sulfate–polyacrylamide gel electrophoresis (SDS-PAGE). After electrophoresis, proteins were transferred to polyvinylidene difluoride (PVDF) membrane (Millipore) and blocked with 5% fat-free dry milk in 1 × Tris-buffered saline (TBST) for 2 h at room temperature. Membranes were then probed with corresponding antibodies at 4°C overnight. Cleaved caspase-3 (#9661), caspase-3 (#9662), cleaved caspase-9 (#9505), caspase-9 (#9508), phospho-cdc2 (#4539), and cdc2 (#77055) antibodies were obtained from Cell Signaling Technology (Danvers, MA). Expression of human β-actin was detected with anti-β-actin antibody (Proteintech, Rosemont, IL) as a loading control. The membranes were then incubated with horseradish peroxidase (HRP)-conjugated secondary antibody (Santa Cruz, CA) and visualized with enhanced chemiluminescence (ECL)-detecting reagents (ComWin Biotech Co., Beijing, China).

### Statistical Analysis

Results were expressed as mean of ≥2 independent experiments with SD values < 10% of the mean. SPSS 19.0 software was used in the statistical analysis, and the means between two groups were compared by one-way analysis of variance (ANOVA) with Dunnett’s test. Values of *P* < 0.05 were considered to indicate significant differences.

## Data Availability

The raw data supporting the conclusions of this manuscript will be made available by the authors, without undue reservation, to any qualified researcher.

## Author Contributions

WS and LeZ designed the project. XL and CC performed the enzymatic screening; YC synthesized the molecules; WH, LHZ and QX performed the in vitro antitumor experiments. LHZ, QJ and LiZ analyzed the data and wrote the manuscript.

## Funding

This work was supported by National Natural Science Foundation of China (Youth Found, Grant No. 81803343) and Natural Science Foundation of Shandong Province (Youth Found, Grant No. ZR2019QH005).

## Conflict of Interest Statement

The authors declare that the research was conducted in the absence of any commercial or financial relationships that could be construed as a potential conflict of interest.
